# Strong social disparities in access to IVF/ICSI despite free cost of treatment: a French population-based nationwide cohort study

**DOI:** 10.1186/s12905-023-02784-4

**Published:** 2023-11-22

**Authors:** Khaoula Ben Messaoud, Juliette Guibert, Jean Bouyer, Elise de La Rochebrochard

**Affiliations:** 1 Institut National d’Etudes Démographiques (Ined), UR14 - Sexual and Reproductive Health and Rights Unit, 93300 Aubervilliers, France; 2 Université Paris-Saclay, UVSQ, Inserm, CESP, 94807 Villejuif, France; 3 Centre Médico-Chirurgical de La Baie de Morlaix, Rond-Point de La Vierge Noire, 29600 Morlaix, France

**Keywords:** Assisted reproduction technologies, Health services accessibility, Socioeconomic disparities, Infertility, Nationwide study, IVF/ ICSI, Clomiphene citrate, Gonadotropins

## Abstract

**Background:**

Access to IVF/ICSI is facilitated when the financial barrier is removed. In a national context where in vitro fertilisation (IVF)/intracytoplasmic sperm Injection (ICSI) treatment is cost-free, how many women do not access IVF/ICSI and what are the factors associated with non-access?

**Methods:**

Using French national health insurance databases, the cohort included 20,240 women aged 18–43 years living in France who underwent unsuccessful treatment (no pregnancy) with clomiphene citrate (CC) and/or gonadotropins with treatment started between January and August 2016. The outcome measure was non-access to IVF/ICSI during the 24-month following start of infertility care. Factors associated with non-access to IVF/ICSI were explored using mixed effects logistic regression.

**Results:**

In the cohort, 65.4% of women did not access IVF/ICSI. In multivariable analysis, non-access to IVF/ICSI was higher in younger women (18–25 years: (OR 2.17, 95% CI: 1.85–2.54) and in older women (40–43 years: (OR=3.60, 95% CI: 3.25–3.98)). Non-access was higher among women below the poverty line (OR=3.76, 95% CI: 3.34–4.23) and showed a significant upward trend with increasing deprivation of place of residence. Distance to the nearest fertility centre was not significantly associated with non-access to IVF/ICSI.

**Conclusions:**

In a national context of cost-free ART treatment, a large proportion of women did not access treatment, with a strong social gradient that raises important issues. We need to understand the underlying social mechanisms to develop an efficient and equitable health policy regarding infertility care.

## Background

Access to assisted reproduction technologies (ART) may be prevented by their high cost [1–4]. Some studies carried out in Canada, the United States and Australia have shown the importance of economic barriers in access to in vitro fertilisation/intracytoplasmic sperm injection (IVF/ICSI) by demonstrating that use of IVF/ICSI increased when health insurance coverage or low-cost programmes were offered [5–9]. During the first year that universal coverage of IVF/ICSI was introduced in Quebec, the number of procedures increased by 192% [7]. Access to ART is probably greatly facilitated in countries such as France, where national health insurance fully covers ART for all women, including those below the poverty line, until their 43rd birthday. However, even in such a favourable national context, barriers to ART access may still exist. Studies have shown an increased risk of non-access among less socially advantaged populations and among populations living in remote areas far from fertility centres [6, 10, 11].

Infertile couples are rarely directly treated by IVF/ICSI in France, like in the USA [12, 13]. Nine in 10 infertile couples first receive a much less invasive and complex treatment based on hormonal drugs, clomiphene citrate (CC) and/or gonadotropins alone, known as ovulation induction [13, 14]. In infertility treatment, IVF/ICSI is often a second-line treatment after ovulation induction by CC and/or gonadotropins has failed.

Our aim was to measure non-access to ART and to identify associated factors among women unsuccessfully treated by CC and/or gonadotropins.

## Methods

### Data source and inclusion criteria

In France, national health insurance covers 98% of the resident population. Its databases exhaustively record all individual drug reimbursements, medical devices, laboratory tests, medical procedures, private and public hospital stays and healthcare. They also provide some information on the patient: sex, age, commune of residence and registration with health insurance for low-income patients. This data source has been described in detail elsewhere [15]. Access to French health insurance databases is subject to strict national legislation and is granted only by a personal time-limited authorisation for a specific research project.

Criteria of inclusion in the cohort study were being a woman living in mainland France, aged 18–43 years, having started CC and/or gonadotropin treatment between 1 January and 31 August 2016, and whose treatment was unsuccessful (i.e. no birth or pregnancy leading to ultrasound monitoring). All women were followed up for 24 months after the start of ovulation induction.

### Outcomes and factors

The outcome of interest was non-access to IVF and ICSI, measured as absence of IVF and ICSI during the 24-month follow-up.

The woman’s age was the age reached by the woman during the year that ovulation induction was started. Social disadvantage (yes/no) was assessed by registration with the health insurance for low-income patients. This indicates that the annual household income is below the poverty line (i.e. < 50% of median annual household income). A complementary social indicator was used measuring the level of deprivation in the patient’s commune of residence, or residence deprivation index [16]. This validated index combines four indicators measured at the level of the commune (local administrative unit) based on data from the 2013 French census (percentage of unemployed individuals, percentage of workers in the active population, percentage of individuals aged 15 and over with high-school graduation and median income per household).

Driving time by road between the commune of residence and the nearest fertility centre was calculated using the tool developed by the French Biomedicine Agency which monitors ART and by the French Institute for Research and Information in Health Economics. It is based on the French road dataset (French National Geographic Institute BD TOPO®) and is weighted by population density and land use.

### Statistical analysis

The risk of non-access to IVF/ICSI was analysed using bivariate and multivariable mixed effect logistic regressions to take into account some clustering in the data for the women’s commune of residence [17, 18]. We reported non-adjusted and adjusted odds ratios (OR), 95% confidence intervals (CI) and *P* values. All statistical analyses were performed using SAS Enterprise Guide software (version 4.3, SAS Institute Inc., Cary, NC, USA). The GLIMMIX procedure was used to estimate OR and 95% CI.

## Results

Our study cohort included 20,240 French women aged 18–43 years unsuccessfully treated by ovulation induction. The median age of the women in this cohort was 34 years and 46.1% were aged 35 years or older. Fourteen percent were socially disadvantaged and 11.6% lived at least 60 min drive from the nearest fertility centre. The global proportion of women who did not access IVF/ICSI treatment within 24 months after the start of ovulation induction was 65.4%, i.e. only 34.6% accessed IVF/ICSI. More than four out of five women did not access IVF/ICSI among socially disadvantaged women (87.2%) and among women aged 40–43 (82.7%) (Table 1).

**Table 1:** Characteristics of the cohort study (*n* = 20,240)

Characteristics	Distribution %	N
**Age (years)**		
**18–24**	5.35	1,083
**25–29**	19.49	3,945
**30–34**	29.10	5,889
**35–39**	27.71	5,609
**40–43**	18.35	3,714
**Below the poverty line**		
**No**	85.98	17,403
**Yes**	14.02	2,837
**Residence deprivation index**		
**Richest fifth**	22.80	4,614
**2nd quintile**	19.94	4,036
**3rd quintile**	18.70	3,785
**4th quintile**	17.94	3,632
**Poorest fifth**	20.62	4,173
**Driving time to the nearest fertility centre**		
**< 30 min**	58.19	11,777
**[30;60[ min**	30.21	6,114
**≥ 60 min**	11.61	2,349

Factors associated with non-access to IVF/ICSI were similar in univariate and multivariate mixed effect logistic regression (Table 2). The woman's age was associated with non-access to ART according to a U-shaped curve: risk of non-access was lowest among women aged 30–34 years and higher among younger and older women. The adjusted odds ratio of non-access to IVF/ICSI was (OR = 2.17, 95% CI: 1.85–2.54) for women aged 18–24, (OR = 1.23, 95% CI: 1.13–1.33) for ages 25–29, (OR = 1.29, 95% CI: 1.19–1.39) for ages 35–39 and (OR = 3.60, 95% CI: 3.25–3.98) for ages 40–43 compared with women aged 30–34 years (P < 0.001). Non-access to ART was higher among socially disadvantaged women with an adjusted odds ratio of (OR = 3.76, 95% CI: 3.34–4.23) compared with non-disadvantaged women. Non-access to IVF/ICSI showed a significant upward trend with increasing deprivation of place of residence, with adjusted odds ratios of non-access to IVF/ICSI increasing from (OR = 1.15, 95% CI: 1.03–1.27) in the 2^nd^ richest fifth, (OR = 1.26, 95% CI: 1.13–1.41) in the 3^rd^ fifth, (OR = 1.33, 95% CI: 1.19–1.49) in the 4^th^ fifth to (OR = 1.60, 95% CI: 1.43–1.78) in the poorest fifth compared with the richest fifth (P < 0.001). Driving time to the nearest fertility centre was not significantly associated with IVF/ICSI non-access with an adjusted odds ratio of (OR = 0.99, 95% CI: 0.92–1.07) for driving time to the nearest fertility centre of 30 to 60 min and (OR = 1.04, 95% CI: 0.93–1.16) for driving time of 60 min or more compared with less than 30 min (*P* = 0.70).

**Table 2:** Non-access to IVF/ ICSI during the 24-month period after the start of ovulation induction by CC and/or gonadotropins among included in the cohort (*n*=20,240); univariate and multivariable analysis^a^

Characteristics	Non-access to ART	Univariable analysis^a^				Multivariable analysis^a^			
		**OR**	**95% CI**		***P*-value**	**OR**	**95% CI**		***P*-value**
**Age (years)**					< 0.001				< 0.001
**18–24**	77.75	2.58	[2.21;	3.01]		2.17	[1.85;	2.54]	
**25–29**	61.85	1.23	[1.13;	1.33]		1.17	[1.07;	1.27]	
**30–34**	56.83	1				1			
**35–39**	63.06	1.29	[1.19;	1.39]		1.30	[1.20;	1.40]	
**40–43**	82.66	3.57	[3.23;	3.95]		3.60	[3.25;	3.98]	
**Below the poverty line**					< 0.001				< 0.001
**No**	61.84	1				1			
**Yes**	87.20	4.06	[3.61;	4.56]		3.76	[3.34;	4.23]	
**Residence deprivation index**					< 0.001				< 0.001
**Richest fifth**	59.93	1				1			
**2nd quintile**	62.49	1.11	[1.00;	1.24]		1.15	[1.03;	1.27]	
**3rd quintile**	65.42	1.24	[1.11;	1.38]		1.26	[1.13;	1.41]	
**4th quintile**	66.82	1.34	[1.20;	1.49]		1.33	[1.19;	1.49]	
**Poorest fifth**	72.99	1.79	[1.61;	2.00]		1.60	[1.43;	1.78]	
**Driving time to the nearest fertility centre**					0.45				0.70
**< 30 min**	65.68	1				1			
**[30;60[ min**	64.83	1.01	[0.93;	1.09]		0.99	[0.92;	1.07]	
**≥ 60 min**	65.43	1.07	[0.96;	1.19]		1.04	[0.93	1.16]	

^a^From univariate and multivariable mixed effect logistic regression models

## Discussion

After unsuccessful treatment by ovulation induction, only one out of three women (34.6%) accessed IVF/ICSI. This demonstrates that non-access to IVF/ICSI is very frequent, even in a context of full health insurance coverage as in France.

Previously, a Dutch study explored the infertility treatment care of women with WHO-II anovulation, i.e. normogonadotropic normoestrogenic anovulation. Of 104 Dutch women unsuccessfully treated with ovulation induction, 53% accessed IVF/ICSI and 47% did not in the following 36-month period [19]. This percentage is well below the 65.4% level of non-access observed in our study, but it remains extremely high considering the longer follow-up (36 versus 24 months). More importantly, these women were in a particularly favourable context to access IVF/ICSI since they had already had ovulation induction in a fertility centre and so did not need to seek another centre to pursue their treatment. We have not identified any literature on this particular point, and it would be interesting to examine whether similar results have been observed in other countries.

Other studies have investigated other stages of infertility treatment care and have also found high levels of discontinuation. As many as 25% to 50% of women do not pursue IVF/ICSI after one or two unsuccessful IVF/ICSI cycles [20, 21], probably in part because of the psychological burden of these treatments [22]. Even before ART, 2 out of 5 infertile couples do not access infertility care in France and in the United States [23, 24]. Our study therefore complements a coherent body of knowledge showing a surprisingly high level of non-access and drop-out at each stage of infertility treatment care. As infertility treatments are fully reimbursed in France, non-access to IVF/ICSI could be even higher in populations with less favourable insurance coverage.

Women aged over 35 years, who made up about half of our cohort (46.1%), faced a higher risk of non-access to IVF/ICSI, and women over 40 years in particular. Since it is very well established that IVF/ICSI success rates decline after the age of 35 years and even more sharply after 40 years [25–27], it can be hypothesized that gynaecologists and ART specialists may tend to discourage or refuse to treat older women, especially if infertility check-up revealed a low ovarian reserve [28, 29]. At the opposite end of the age scale, the few young women in our study (5.4% aged 18–24 years) also had a higher risk of non-access to IVF/ICSI. It would be useful to investigate the characteristics of these young women and the rationale of their medical care.

Even though in France infertility treatments are fully reimbursed, women who were socially disadvantaged or living in a deprived area had a higher risk of non-access to IVF/ICSI than more advantaged women. This result is in line with a US study of women with low annual incomes who benefited from complete coverage of IVF/ICSI treatments [30]. Another US study pointed out that the social gradient may partly reflect difficulties in finding a doctor with whom disadvantaged women felt comfortable, as well as difficulties in taking time off for the multiple appointments needed during infertility treatment [31]. More broadly, studies have suggested potential social barriers such as difficulties in communicating with medical providers, the indirect cost of time-consuming infertility treatment, the complexity and bureaucracy of the ART process, and the stigma attached to infertility that may be stronger depending on cultural background [32–35].

Our finding that driving time to the nearest fertility center was not significantly associated with non-access to IVF/ICSI is in line with a US study [36]. However, an Australian study concluded on an increased risk of non-access to IVF/ICSI in remote areas compared with major cities [11]. This latter result may be explained by a very low population density in Australia and much longer distances to remote areas.

Using French health insurance databases, access to assisted reproduction techniques was investigated for the first time in the general population by considering all national fertility centres. Unlike our study, previous work has investigated access to or pursuit of IVF/ICSI based only on patients recruited in specific fertility centres [33, 37, 38]. Moreover, these earlier studies were not able to distinguish between non-access and treatment in another fertility centre [39].

This study was limited by the lack of information on causes of infertility, duration of infertility or parity. It was also limited by the lack of the indication on the reasons for non-access to IVF/ICSI that could be due to barriers to access but also to medical reasons, the decision to access cross-border reproductive care or to choose adoption, or to abandon the project of having a child [40]. Finally, only infertile women treated by ovulation induction (CC and/or gonadotropins) were included in this study. Nevertheless, this represents 9 of 10 infertile women in France [13].

## Conclusions

Our findings suggest that several mechanisms are involved in the very high level of non-access to IVF/ICSI even when care is fully covered by health insurance. Firstly, an expected low probability of successful IVF/ICSI may play a part in older women, either through a medical decision or a mutual patient-doctor decision not to treat. This decision process deserves further investigation. Secondly, we observed that a strong social gradient affected access to IVF/ICSI. Crucially, we need to understand the underlying social mechanisms. Possibly, IVF/ICSI may be less accepted among socially disadvantaged patients and/or may be more difficult to reconcile with their daily work constraints. Moreover, socially disadvantaged patients may find it more difficult to interact with ART specialists, either because these specialists do not sufficiently adapt to these patients or because they (unconsciously) discriminate against them [41]. To unravel the mechanisms of social barriers to IVF/ICSI access would be of primary importance in developing an efficient and equitable health policy regarding infertility care.

## Data Availability

To request access to French health insurance database, please contact the French health Data Hub (website: https://www.health-data-hub.fr/) in accordance with French law (National Decree no. 2021–848, 29 June 2021). The SAS code underlying this article will be shared on reasonable request to the corresponding author.

## Abbreviations

IVF: In vitro fertilisation
ICSI: Intracytoplasmic sperm injection
CC: Clomiphene citrate
ART: Assisted reproduction technologies
